# MRI Phantoms – Are There Alternatives to Agar?

**DOI:** 10.1371/journal.pone.0070343

**Published:** 2013-08-05

**Authors:** Alexandra Hellerbach, Verena Schuster, Andreas Jansen, Jens Sommer

**Affiliations:** Department of Psychiatry and Psychotherapy, Philipps-University Marburg, Marburg, Germany; University Hospital La Paz, Spain

## Abstract

The suitability of different gelling agents as MRI phantoms was evaluated in terms of homogeneity, gel stability and reproducibility. Time and effort for preparation were also taken into account. The relaxation times of various gel compositions were estimated. Carbomer-980 and Carbopol-974P were determined to be promising novel phantom materials. These gelling agents are readily available, inexpensive and easy to handle given that thermal treatment is not required. Furthermore, the viscoelasticity of their polymer network is pH-dependent. With such characteristics, it was even possible to embed sensitive objects and retrieve them after testing. This was demonstrated with a fiber phantom for Diffusion Weighted MRI applications. Since Carbomer-980 and Carbopol-974P are non-hazardous, they are also suitable for multimodal setups (e.g., MRI as well as ultrasonic imaging).

## Introduction

Over the last 25 years, magnetic resonance imaging (MRI) has become a major diagnostic tool in clinical imaging and scientific research. Although modern systems show good technical quality (i.e. high signal-to-noise ratio, good image homogeneity and minimal ghosting) and differentiation between tissue classes (i.e. image contrast), absolute signal intensities of acquired images are not reproducible between systems of the same or different make and type [1], [2]. Image values and contrast are dependent on the sequence parameter settings and on MR scanner properties [2]. Additionally MRI artefacts, such as intensity non-uniformity, introduce spatial variations in the image intensity. Magnetic field inhomogeneities or fluctuations in SNR may cause intensity variations. This might be a problem for longitudinal studies as changes in acquisition protocols and scanner upgrades may lead to inaccuracies of measurements over time [2]. Especially when MRI data is collected at multiple sites or with several different scanners, vendor-specific differences in scanner and pulse sequence characteristics should be taken into account.

MRI multicenter studies are becoming increasingly more common in order to obtain large study cohorts that are sufficiently powered for statistical analysis [2], [3]. To ensure consistent MRI interpretation at participating facilities, it is essential to have a means for system comparison beyond basic imaging parameters. Specifically, there is a need for an MRI phantom suitable for quality assurance purposes, but also for testing instrument performance and evaluating new imaging techniques and sequences. Since such a multi-purpose phantom is not feasible [3], [4], [5], we sought to determine the most ideal material to quickly and easily prepare phantoms.

Established test objects in the field of quality assurance (QA) are the MRI phantom of the American College of Radiology (ACR) [4], [6] and the agar gel phantom proposed by the Functional Bioinformatics Research Network (FBIRN)- Consortium [3]. Whereas the ACR phantom is well suited for testing system performance, the FBIRN phantom was developed for QA of functional studies.

In the field of Diffusion Tensor Imaging (DTI) special fiber phantoms with anisotropic characteristics are necessary. In this article we focus on the embedding material for such fiber phantoms.

Desired characteristics of the phantom material include: 1) comparable relaxation times to those of human tissue, 2) robustly processable, 3) non-hazardous, 4) stable for long periods of time, 5) readily available, 6) inexpensive, and 7) easy to handle.

Although many different types of phantom materials have been proposed, water, agar and agarose are the most commonly used [7], . Occasional use of polyvinyl alcohol (PVA) [12], [13], gelatin 14,15, TX-150 [16], TX-151 [17] and carrageenan [18], [19] has also been reported. Water phantoms are easy and safe to handle, but require a 10 min settling time. Moreover, water is easily influenced by vibrational effects.

With regards to preparation of more complex phantoms, such as diffusion phantoms, embedding fiber objects in a gel reduces artifacts on boundaries and avoids water flow. Gel preparation without thermal treatment is preferable to ensure that fiber object properties are not altered during the sealing process. Complexity of the preparation process, especially a thermal treatment step, is dependent on the type of phantom material and the intended quality of the resulting gel. For production of PVA gel, a series of freeze-thaw cycles of the aqueous PVA solution is required [13]. A similarly laborious step, degassing of water at approximately 10°C [17], is required for polysaccharide gel preparation of TX-151. Most other polysaccharides, such as agar, agarose or carrageenan, need high thermal treatment (80–100°C) during the preparation process.

T_2_ relaxation time of both agarose and agar gel is similar to that of human tissue (40–150 ms), and can be adjusted by altering the consistency of the gel (i.e. the concentration of agarose or agar) [9], [11]. On the contrary T_1_ relaxation time is not comparable to that of human tissue. For modification of T_1_ relaxation time, gel phantoms usually contain paramagnetic additives, such as gadolinium chloride or nickel chloride [10]. Additionally agarose gel phantoms often contain sodium azide to retard mold formation [3]. Toxic additives such as nickel chloride and sodium azide slow mold growth and may modulate relaxation times, but the handling of phantoms prepared with toxic additives is complicated. Shipping and disposal of toxic phantoms would also be problematic, and broken phantoms could lead to contamination of MRI instrumentation, staff and researchers. Another disadvantage of using agar and agarose gel as phantom material is the fact that agarose is a natural product. Therefore gel and relaxation properties may differ somewhat between different product batches. In contrast the production of synthetic polymers would be supremely standardized.

In the current study, 6 different gelling agents were evaluated to determine which could potentially be used as an optimal phantom material, providing an adequate alternative to agar gels.

## Materials and Methods

### Choice of Gelling Agents

The gelling properties of sodium alginate (E401, BaccaraRose, Germany), xanthan gum (BaccaraRose, Germany), FAVOR-PAC-300 (Evonik Industries AG, Germany), PNC-400 (FRAGON GmbH et Co. KG, Germany), Carbomer-980 (Euro OTC Pharma GmbH, Germany) and Carbopol-974P (Euro OTC Pharma GmbH, Germany) were evaluated. Since these gelling agents are common in the pharmaceutical, cosmetic and food industries, they are part of everyday living.

Alginate is an anionic polysaccharide extracted from the cell wall of brown algae. It consists of homopolymeric blocks of uronic acid. Sodium alginate (NaC_6_H_7_O_6_) is the sodium salt of alginic acid. It is a well-known food additive and very popular in molecular gastronomy.

Xanthan gum is a polysaccharide produced by fermentation of glucose, sucrose or lactose by Xanthomonas bacteria. It is used in the cosmetic, food and oil industries as a thickening agent. It is highly stable under a wide range of temperatures and pH values, but high shear rates can compromise gel properties (elasticity, viscosity).

FAVOR-PAC is a product line of superabsorbent granules that have been specifically designed for the packaging industry. It is an expandable, cross-linked polymer that can absorb and retain large amounts of liquid. Total absorbance capacity is dependent on the ionic concentration of the aqueous solution and the degree of cross-linking in the polymer. In deionized and distilled water, PAC-300 can absorb 500–1000 times its own weight, whereas absorbency drops by factor of ten in a physiological saline solution. Polymers containing extensive cross-linking have a lower absorbance capacity than low density cross-linking polymers, but gel strength and shape maintenance are superior.

PNC-400, also referred to as sodium polyacrylate or sodium carbomer, is also a type of superabsorbent polymer. The chemical formula is [CH_2_-CH(COONa)]_n_). PNC-400 is synthesized by polymerization of acrylic acid blended with sodium hydroxide and UV initiator to form a polyacrylic acid sodium salt. Synthetic production of polyacrylate co-polymers guarantees long term stability. Two other types of polyacrylic acids are Carbomer-980 and Carbopol-974P. All three types of polyacrylic acids have similar chemical properties. Carbomer code is an indicator of molecular weight and gel viscosity of homopolymers. The type and density of homopolymers may influence gel strength, homogeneity and imaging characteristics.

### Sample Preparation

A series of test gels were prepared by mixing the agents with distilled water (50 ml) at room temperature. A magnetic stirrer was used to achieve uniform distribution. Concentrations of gelling agents ranged from 0.5% to 5.0%. Gels were prepared in small open glass vessels to examine susceptibility to fungal growth. A second group of test phantoms was created in 500 ml propylene containers. A third and final group with different contrast agents was prepared for analyzing variations in relaxation times. Gadolinium-based contrast agents, such as Magnevist (Bayer HealthCare) and Primovist (Bayer HealthCare), were used as T_1_ modifiers. For modification of T_2_ relaxation time, ferric chloride, manganese nitrate and Endorem (a super paramagnetic contrast agent; Guerbet) were applied. Flucytosine (5-FC) was used as an antiseptic, but can also affect T_1_ and T_2_ relaxation times. Effect of pH and powder concentration on relaxation times was analyzed using Carbomer-980 test gels. All samples were prepared using the following procedure: 1) pre-measuring all components (e.g. 2.5 g Cabomer-980 powder, 500 ml distilled water and 0.017 g manganese nitrate powder when necessary to modify relaxation time), 2) resolving contrast agent into water and mixing the solution with polymer powder until completely dissolved, 3) adding sodium hydroxide neutralizer to create a solid gel (in case of Carbomer-980 and Carbopol-974P), and 4) sealing the sample with PARAFILM® M (Carl Roth, Germany).

### DTI Phantom Preparation

A DTI phantom was created by winding 15 µm polyester fibers (Filament garn dtex 76f32 Trevira GmbH) around a cylindrical polyamide spindle. The fibers were soaked in a sodium chloride solution during the winding process to minimize magnetic susceptibility differences between fluid and fibers [20]. Phantom winding was performed using an in-house winding machine. Fiber phantoms were sealed with latex milk after the winding process. The DTI phantom was first measured in water (embedded in wadding to reduce water movements) then sealed into a gel.

### Image Acquisition and Analysis

Data was acquired using a 3T MR scanner (Magnetom Trio, Siemens Medical Solutions, Erlangen) and a 12-channel head coil for reception. Measurements of T_1_-weighted images were performed using a three-dimensional gradient-echo sequence (MP RAGE) with an isometric voxel size of 1×1×1 mm^3^. Other acquisition parameters were: TR of 1500 ms, TI of 900 ms, TE of 2.52 ms and matrix size of 152×256. Measurements of T_2_-weighted images were performed using a three-dimensional turbo spin echo (TSE) sequence with slab selective, variable excitation pulse (SPACE). Acquisition parameters were: isometric voxel size (1×1×1 mm^3^), matrix size = 153×256, TE = 402 ms and TR = 3000 ms. Relaxometry measurements were made using the spin-echo method [21]. For evaluation of T_1_ relaxation time, a series of measurements were performed using a TE of 20 ms with TR varying from 1500 ms to 5000 ms. Other acquisition parameters were: slice thickness = 6 mm, FOV = 200×200 and matrix size of 128×33. For evaluation of T_2_ relaxation time, TE was varied from 10 ms to 200 ms with a TR of 3000 ms. Other acquisition parameters were: slice thickness = 6 mm, FOV = 200×200 and matrix size = 128×33. A ROI-based approach was used to determine T_1_ and T_2_ relaxation times. Based on acquired images, the mean signal and standard deviation of each measurement from a region of interest, which covered 75% of the phantom image, were calculated. The ROI was positioned in the center of the image and contains at least 1000 pixels or 75% of the phantom area. An area of only 75% of the phantom image was taken into account to avoid any edge artefacts. For a spin-echo sequence, MR signal is calculated using [22]

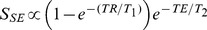
(1)where T_1_ and T_2_ relaxation times are estimated by fitting the mean signal values of the defined ROIs to the corresponding mono-exponential equations.

For comparison of fiber phantom measurements in gel and aqueous solution, DTI data was acquired with 30 gradient directions and an isometric voxel size of 2×2×2 mm^3^. A b-value of zero and 700 s/mm^2^ were used. Other DTI acquisition parameters were TR = 7800 ms, TE = 90 ms, axial slice number of 50, phase-encoding direction A–P and parallel imaging with GRAPPA (set to 2). For estimation of FA maps DTIFIT from FSL (FMRIB Software Library, Oxford) was used.

## Results

### Test Gels Characteristics

**Figure 1:**
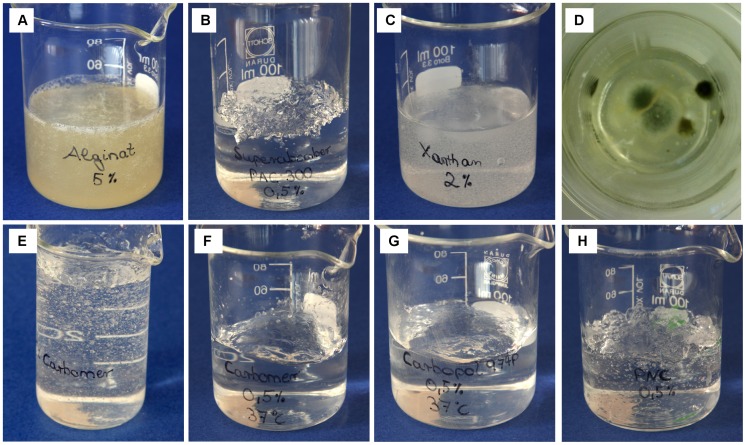
Test gels. **A** sodium alginate gel, **B** PAC-300 gel, **C** xanthan gel, **D** xanthan gel with mold growth after 2 weeks, **E** Carbomer-980 gel, **F** Carbomer-980 gel prepared with warm water (37°C, shows less bubble formation and better homogeneity). **G** Carbopol-974P gel prepared with warm water (37°C, no bubbles visible). **H** PNC-400 gel prepared with warm water (37°C, bubble formation is still visible).

**Figure 2:**
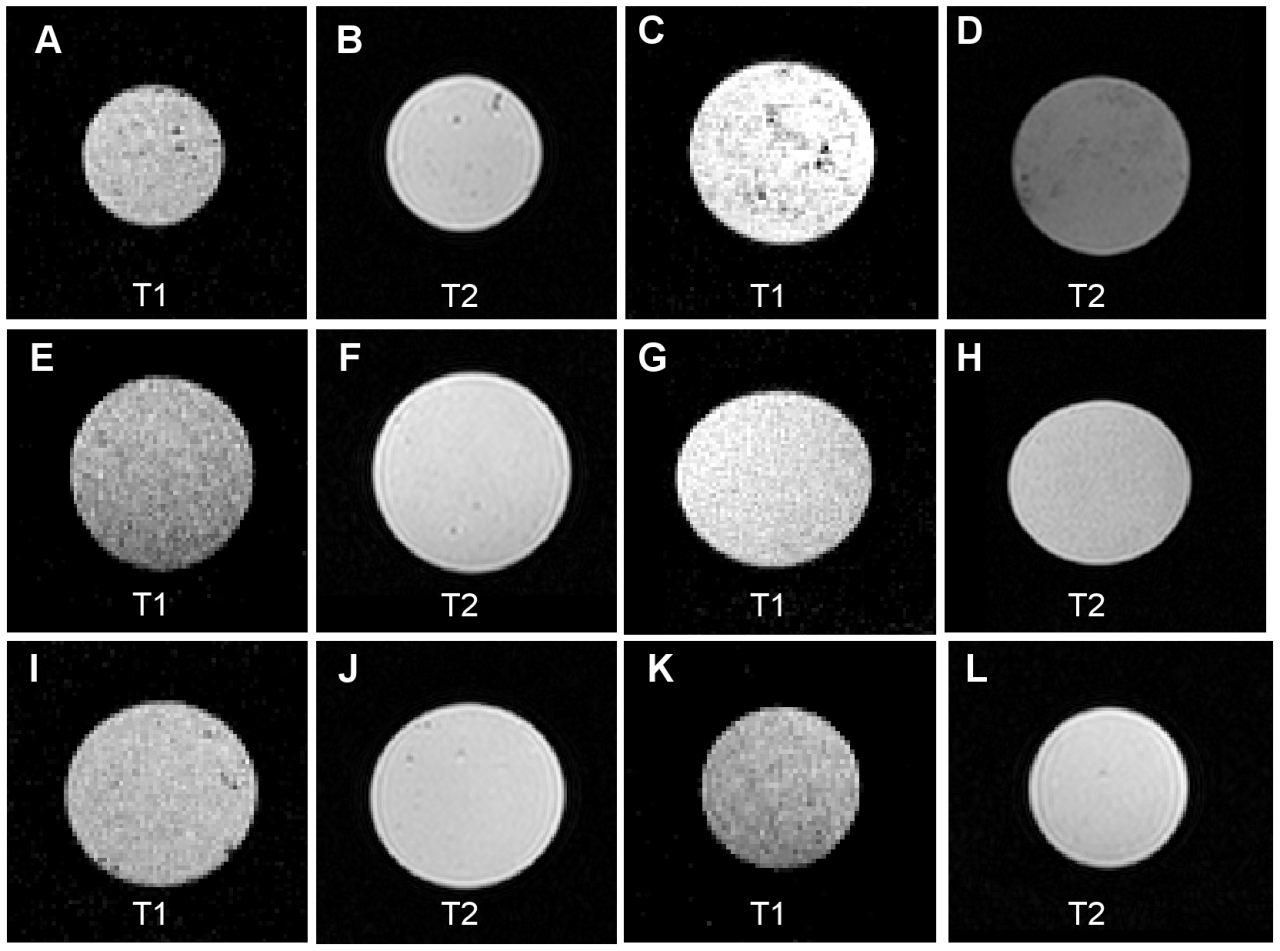
T_1_- and T_2_-weighted images. **A+B** PNC-400 gel, **C+D** sodium alginate gel, **E+F** PAC-300 gel, **G+H** xanthan gel, **I+J** Carbopol-974P gel, **K+L** Carbomer-980 gel.

The prepared test gels are shown in Fig. 1. Measurements of T_1_- and T_2_-weighted images of test gels are shown in Fig. 2. Sodium alginate and xanthan both formed a creamier liquid gel as oppose to a solid gel. Moreover, xanthan was highly susceptible to fungal growth, which was visible after 5 days. Fig. 1D shows mold formation after 2 weeks. Fungal growth was not observed to develop on any other test gels (three-month observation period). PAC-300, PNC-400, Carbopol-974P and Carbomer-980 formed solid transparent gels. Handling of PAC-300 gel seemed to lead to deterioration of gel strength. Carbopol-974P and Carbomer-980 polymers had to be neutralized to achieve maximum gel viscosity because gelation process of polyacrylic acids (e.g. Carbomer-980 and Carbopol-974P) is pH-dependent. Gelation of Carbomer-980 and Carbopol-974P gels has started at a pH of about 4.0. Non-neutralized polymer solution of Carbomer-980 and Carbopol-974P had an approximate pH of 2.0–3.0 depending on polymer concentration. Sodium hydroxide was used as neutralizer for gel preparation of Carbomer-980 and Carbopol-974P gels. Without adding a neutralizer, both Carbopol-974P and Carbomer-980 gels were essentially liquid. Once sodium hydroxide was added, thickening gradually occurred. High viscosities were observed when pH ranged from 5.0–8.0. Gel viscosity began to decrease at pH 8.0 and higher. This can be explained by dampening of electrostatic repulsion caused by the excess of electrolytes at high pH values. Desired gel viscosity can be obtained by adjusting pH, and gelation could be easily reversed for Carbomer-980 and Carbopol-974P gels. Another type of polyacrylic acid is PNC-400. Non-neutralized polymer mixture of PNC-400 had an approximate pH of 6.0–8.0, so gelation process has started instantly. Formation of an abundance of air bubbles, especially using PNC-400, was noted in all test gels. Gel preparation with warm (30°C-40°C) or degassed water will reduce air bubble formation (Fig. 1F–H).

### Relaxation Times of Samples

**Figure 3:**
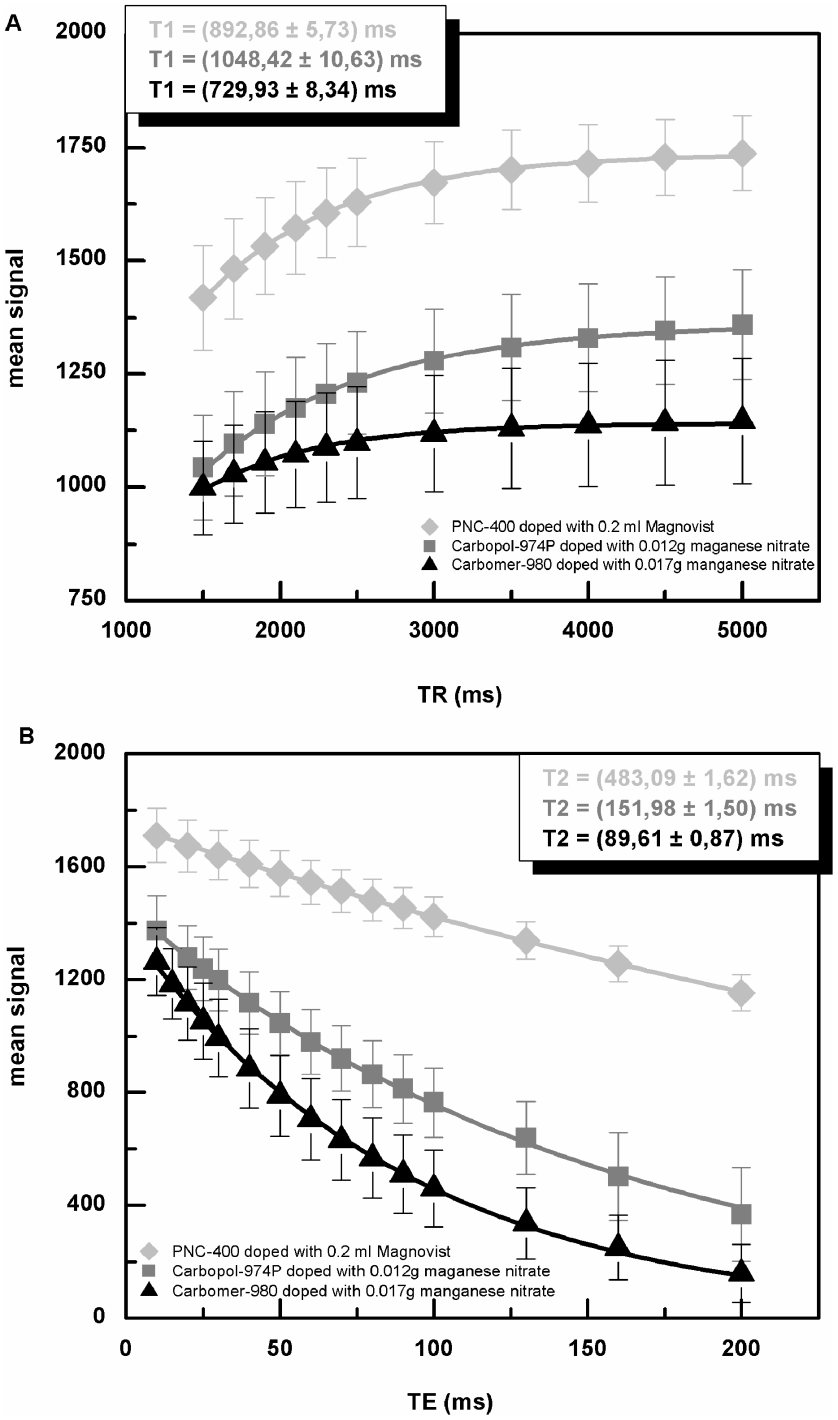
Estimation of T_1_ and T_2_ relaxation times from three samples. **A** Fits of T_1_ relaxation time for PNC-400 phantom mixed with 0.20 ml Magnevist, Carbopol-974P phantom mixed with 0.012 g manganese nitrate and Carbomer-980 phantom mixed with 0.017 g manganese nitrate. **B** Fits of T_2_ relaxation time for PNC-400 phantom mixed with 0.20 ml Magnevist, Carbopol-974P phantom mixed with 0.012 g manganese nitrate and Carbomer-980 phantom mixed with 0.017 g manganese nitrate.

**Figure 4:**
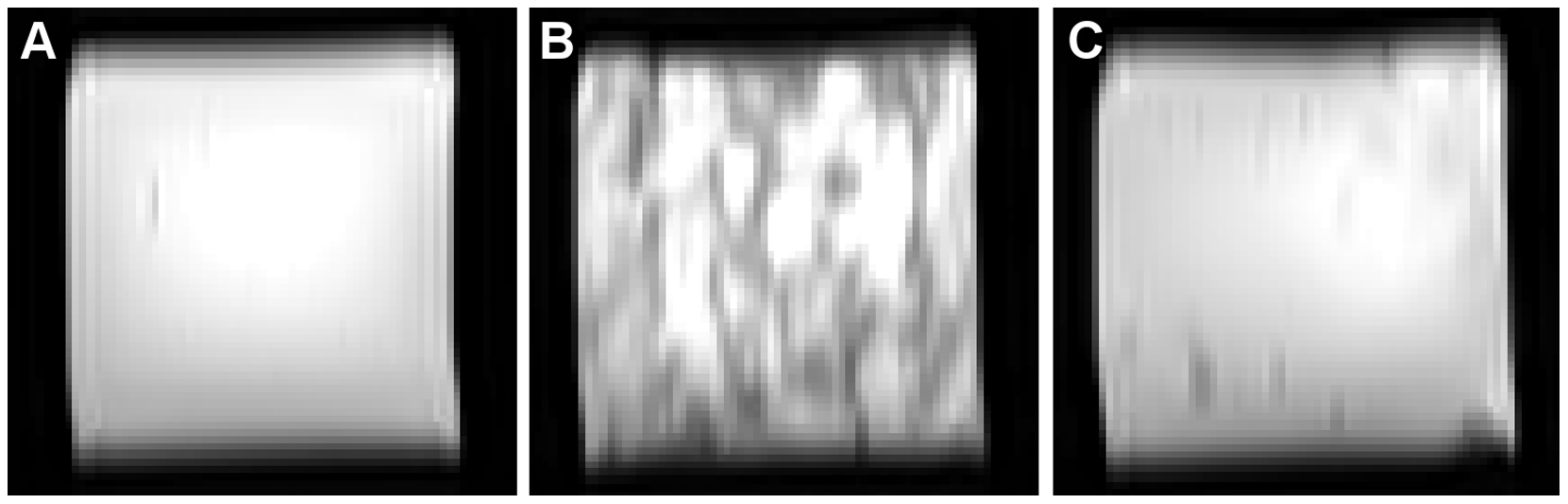
Effect of contrast agents on image homogeneity. T_2_ image of Carbopol-974P phantom (0.5% Carbopol-974P, spin echo sequence with TE = 50 ms and TR = 3000 ms). **A** Phantom without contrast agent. **B** Phantom with 0.20 ml Endorem and **C** phantom with 0.012 g manganese nitrate.

Relaxation times of all gelling agents are listed in Table 1. Sodium alginate had relaxation times similar to those of human tissue, whereas T_1_ and T_2_ relaxation times of other gelling agents were longer than those of human tissue. For comparison T_1_ and T_2_ relaxation times of different biological tissues measured at 37°C and 3 T are presented in Table 2 [23]. Relaxation times could be adjusted by the addition of contrast agents. T_1_ and T_2_ modification in different samples is summarized in Table 3. For analysis of variation in T_1_ and T_2_ relaxation times, only phantoms with a solid gel structure were used. Relaxometry fit curves of three different samples are shown in Fig. 3. Without adding a contrast agent, T_2_ relaxation time was similar to that of free water. Manganese nitrate and Endorem reduced T_2_ relaxation time to a value similar to that obtained from human tissue. Depending on the concentration, T_2_ modifiers had a strong influence on gel homogeneity (Fig. 4). Another way to reduce T_2_ relaxation time was to increase the concentration of the gelling agent. Table 4 shows the dependence of relaxation time on Carbomer-980 concentration. The higher the concentration the lower the T_2_ relaxation time, but concentration higher than 1.5% affects also gel homogeneity. pH had a strong effect on viscosity of Carbomer-980 gels, but a less drastic effect on T_1_ and T_2_ relaxation times (Table 5).

**Table 1:** Relaxation times of six different gelling agents.

Gelling agent	Concentration (%)	T_1_ (ms)	T_2_ (ms)
Sodium alginate	5.0	2214.27±23.93	94.34±1.64
Xanthan	2.0	2055.52±12.30	253.81±6.35
PAC-300	0.5	3348.20±46.11	1688.61±184.29
PNC-400	0.5	4372.04±92.92	1482.38±17.37
Carbomer-980	0.5	4580.29±72.85	1453.29±13.40
Carbopol-974P	0.5	4753.28±77.67	1793.32±16.08

**Table 2:** Relaxation times of biological tissue.

Tissue	T1 (ms)	T2 (ms)
Liver (mouse in vitro)	812±64^(23)^	42±3^(23)^
Heart (mouse in vitro)	1471±31^(23)^	47±11^(23)^
White matter (rat in vitro)	1084±45^(23)^	69±3^(23)^
Gray matter (rat in vitro)	1820±114^(23)^	99±7^(23)^
Human blood	1932±85^(23)^	275±50^(23)^

Literature data of T_1_ and T_2_ relaxation times at 3 T measured at 37° [23].

**Table 3:** Relaxation times of PNC-400 (0.5%), Carbopol-974P (0.5%) and Carbomer-980 (0.5%) mixed with different contrast agents.

Gelling agent	Contrast medium	Amount of contrast medium	T_1_ (ms)	T_2_ (ms)
PNC-400	KM1^1^	0.20 ml	1077.68±12.68	602.41±1.38
PNC-400	KM2^2^	0.20 ml	1195.62±6.12	641.03±1.98
PNC-400	KM1+5FC^3^	0.20 ml +0.030 g	892.86±5.73	483.09±1.62
PNC-400	KM1+KM3^4^	0.20 ml +0.20 ml	1933.72±7.96	74.74±4.06
PNC-400	FeCl_2_	0.150 g	2405.59±14.67	350.88±10.94
Carbopol-974P	KM3	0.20 ml	2460.33±15.54	76.98±1.61
Carbopol-974P	Mn(NO_3_)_2_	0.012 g	1048.42±10.63	151.98±1.50
Carbomer-980	Mn(NO_3_)_2_	0.017 g	729.93±8.34	89.61±0.87
Carbomer-980	Mn(NO_3_)_2_	0.020 g	595.24±9.16	82.03±0.92

1 Magnevist,

2 Primovist,

3 flucytosine,

4 Endorem.

**Table 4:** Relaxation times are dependent on concentration of the Carbomer-980 gel.

Concentration ofCarbomer-980 (%)	NaOH (ml)	pH	T_1_ (ms)	T_2_ (ms)
0.5	1.5	5.5	4580.29±72.85	1453.29±13.40
1.0	1.5	6.3	4414.02±72.65	1273.96±9.60
1.5	1.5	4.6	4388.12±73.41	892.86±9.26

**Table 5:** Relaxation times are dependent on pH of the Carbomer-980 gels (0.5%).

NaOH (ml)	pH	T_1_ (ms)	T_2_ (ms)
1.0	4.7	4471.55±73.53	1619.68±20.57
1.5	5.5	4580.29±72.85	1453.29±13.40
2.5	7.5	4588.33±72.94	1484.21±11.77
5.0	8.0	4475.45±81.08	1436.07±8.42
6.0	10.0	4502.31±75.14	1496.24±20.87

### DTI Phantom Measurements

**Figure 5:**
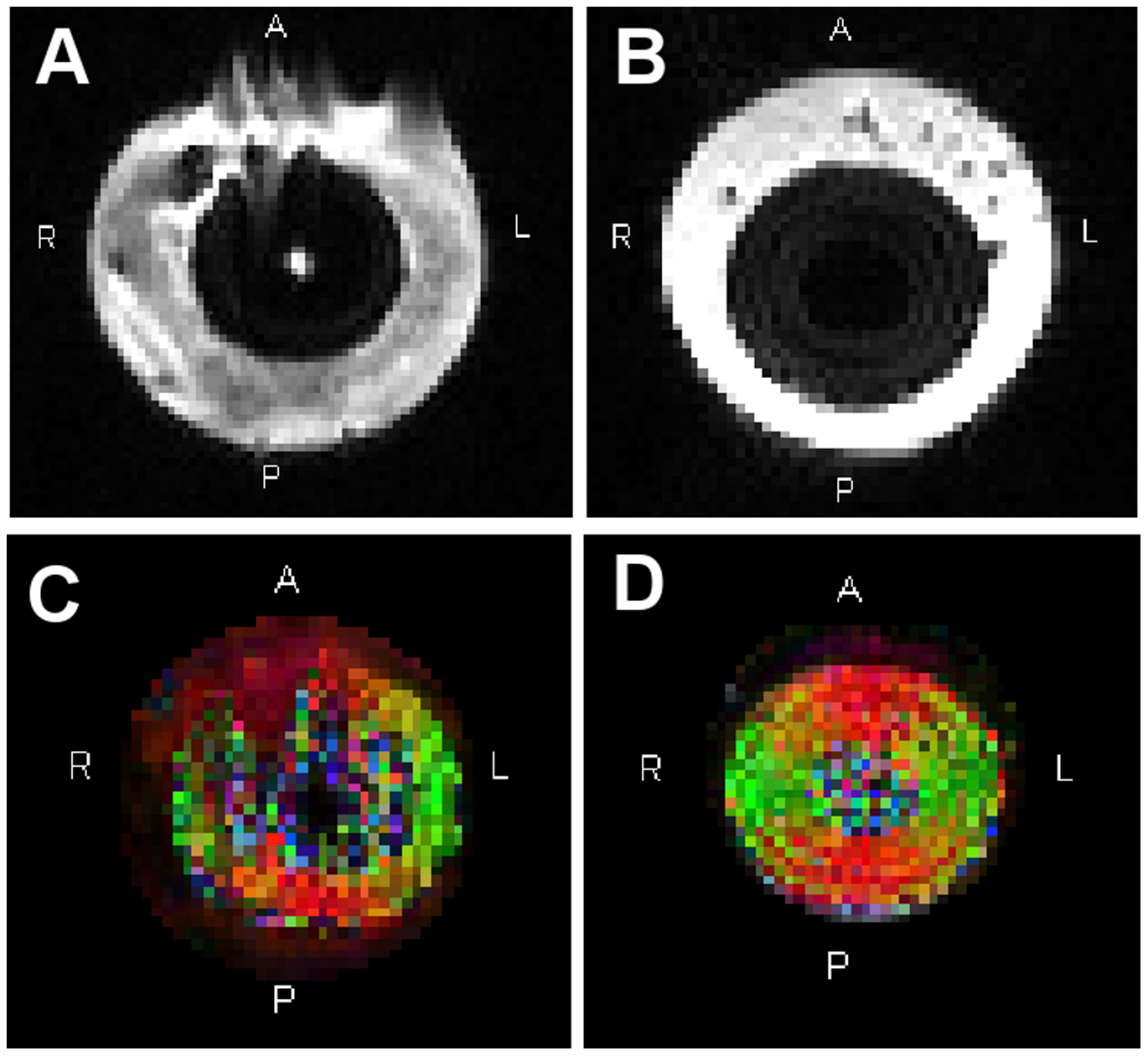
Measurements of the diffusion phantom. **A** b0-image of DTI phantom measured in aqueous solution **B** b0-image of the fiber phantom in Carbomer-980 gel (pH = 13 and concentration = 0.8%). **C** Color-coded FA map of the same DTI phantom measured in aqueous solution and **D** embedded in gel. Phase encoding direction was A–P. Colors indicate direction of polyester fibers. They refer to the standard DTI color scheme (*red*: left- right (x-direction), *green*: anterior – posterior (y-direction), *blue*: inferior-superior (z-direction)).

Fig. 5 shows b0-images and FA maps of the DTI phantom measurements in aqueous solution and when embedded in Carbomer-980 gel. B0-images of the gel phantom showed less distortion compared to measurement in aqueous solution. The fiber phantom and its corresponding FA values were readily identifiable in the gel environment, whereas motion artifacts caused by water movements made correct estimation of FA values difficult when embedded in the aqueous solution.

## Discussion

Gels based on synthetic polymers of acrylic acid (PNC-400, Carbopol-974P and Carbomer-980) show promise as potential alternatives to agar gels. Preparation of the gels did not require special equipment, and the resulting gel was solid and stable for a prolonged period of time. The gelling agents are inexpensive and non-hazardous, and preparation was very straightforward, involving only pre-measurement of water and dry chemical then mixing and final adjustment of pH to obtain desired viscosity. Most notably, there was no need for thermal treatment during the preparation process. Nevertheless, T_1_ and T_2_ relaxation times of the pure materials were longer than those of human tissue.

While it is possible to vary T_2_ relaxation time through altered consistency of the gel (i.e. altered concentration of the gelling agent), only the addition of a contrast agent seems to be able to allow adjustment of T_1_ relaxation time. But if one compares, for example, the T_2_ relaxation time of the pure materials of polyacrylic acids (>1000 ms) with the T_2_ relaxation times of the listed biological tissue (40–300 ms), adaption of T_2_ relaxation time seems to be a little bit more challenging. Altering concentration of the gelling agent allows modification of T_2_ relaxation time only in small increments. Gel preparation becomes more difficult at high concentrations because the powder does not dissolve completely without using auxiliary equipment. For example, using a homogenizer may support the dissolving process and may improve the resulting gel homogeneity. Addition of a contrast agent (e.g. Mn(NO_3_)_2_) enables changing the T_2_ relaxation time in larger increments. The combination of the proposed polyacrylic polymer gels with different types and amounts of contrast agents allow a great diversity of creating phantoms with a large range of relaxation times.

Unfortunately, not all tested gelling agents have advantageous characteristics compared to agar gels. Xanthan gel was just as agar gels highly susceptible to fungal growth, so it would need toxic additives to retard mold formation. Test gels based on sodium alginate did not show a solid gel structure, so they could be easily influenced by vibrational effects.

Regardless, the proposed gelling agents based on synthetic polymers of acrylic acid (PNC-400, Carbopol-974P and Carbomer-980) are easier to handle compared to agar. In particular, since the viscosity of Carbopol-974P and Carbomer-980 is dependent on pH, it is easy to seal fiber objects and retrieve them without thermal or mechanical stress. This was demonstrated with the DTI fiber phantom.

As the proposed gelling materials are non hazardous and allow a straightforward preparation without a thermal treatment step, they extend the options for the creation of more sophisticated phantoms and facilitate the handling of multimodal setups (e.g. MRI and ultrasonic imaging). For example complex phantoms with spatially varying mechanical objects or phantoms which mimic pathological processes (e.g. glioma) can be other areas of interest. In conclusion, the proposed gelling agents may be suitable alternatives to agar.
